# Adult Vampire Bats Produce Contact Calls When Isolated: Acoustic Variation by Species, Population, Colony, and Individual

**DOI:** 10.1371/journal.pone.0038791

**Published:** 2012-06-14

**Authors:** Gerald G. Carter, Ryane Logsdon, Bryan D. Arnold, Angelica Menchaca, Rodrigo A. Medellin

**Affiliations:** 1 Biological Sciences, University of Maryland, College Park, Maryland, United States of America; 2 Organization for Bat Conservation, Bloomfield Hills, Michigan, United States of America; 3 Biology Department, University of Rochester, Rochester, New York, United States of America; 4 Department of Biological Sciences, Murray State University, Murray, Kentucky, United States of America; 5 Instituto de Ecologia, UNAM, Mexico City, Mexico; University of Regina, Canada

## Abstract

**Background:**

Bat pups produce individually distinct isolation calls to facilitate maternal recognition. Increasing evidence suggests that, in group-living bat species, adults often use similar calls to maintain contact. We investigated if isolated adults from all three species of the highly cooperative vampire bats (Phyllostomidae: Desmodontinae) would produce vocally distinct contact calls when physically isolated.

**Methods/Principal Findings:**

We assessed variation in contact calls recorded from isolated captive and wild-caught adult common vampire bats (*Desmodus rotundus*), white-winged vampire bats (*Diaemus youngi*) and hairy-legged vampire bats (*Diphylla ecaudata*). We compared species-typical contact call structure, and used information theory and permuted discriminate function analyses to examine call structure variation, and to determine if the individuality of contact calls is encoded by different call features across species and populations. We found that isolated adult vampire bats produce contact calls that vary by species, population, colony, and individual. However, much variation occurred within a single context and individual. We estimated signature information for captive *Diaemus* (same colony), captive *Desmodus* (same colony), and wild *Desmodus* (different colonies) at 3.21, 3.26, and 3.88 bits, respectively. Contact calls from a captive colony of *Desmodus* were less individually distinct than calls from wild-caught *Desmodus* from different colonies. Both the degree of individuality and parameters encoding individuality differed between the bats from a single captive colony and the wild-caught individuals from different groups. This result is consistent with, but not sufficient evidence of, vocal convergence in groups.

**Conclusion:**

Our results show that adult vampire bats of all three species produce highly variable contact calls when isolated. Contact calls contain sufficient information for vocal discrimination, but also possess more intra-individual variation than is required for the sole purpose of identifying individuals.

## Introduction

Many group-living birds and mammals produce contact calls consisting of a series of harmonically rich, frequency-modulated syllables or notes (e.g. bottlenose dolphins [1], orange-fronted parakeets [2], cotton-top tamarins [3], for other examples see Bradbury & Vehrencamp [4]). Such signals often show “signature” variation [5] and are particularly important for animals that must maintain contact under conditions of low visibility. Echolocating bats live largely in a world of sound. In complete darkness, they perceive their surroundings with biosonar [6]–[7], track conspecifics by eavesdropping [8]–[11], identify offspring [5], [12], and communicate using an extensive repertoire of social calls, which have diverse forms and functions within and between species [12]–[19]. In every bat species studied thus far, non-volant pups produce isolation calls that allow mothers to find and recognize them [12]. Bohn et al. [20]–[21] illustrated the importance of isolation calls by showing correlated evolution between the hearing sensitivities of bats and the frequency of their species-specific isolation calls.

Analysis of animal signals through information theory (bits) allows the comparison of information content across different species, sample sizes, and signal modalities [5]. Wilkinson [12] showed that the information content of isolation calls correlates with colony size across eight species of bats, suggesting that vocal discrimination of pups in large colonies has driven the evolution of isolation call individuality. An illustrative example is provided by Mexican free-tailed bats, *Tadarida brasiliensis*, which produced the most variable isolation calls in his study [5], [12]. Female *T. brasiliensis* raise offspring in what are arguably the densest vertebrate aggregations, where a single cave might be inhabited by more than a million individuals [22], and each lactating female returning from foraging must find her single pup among thousands of others twice per day in total darkness [23]. The estimated complexity of these pup isolation calls at 9 bits [12] allows for 512 unique signature calls. In contrast, the isolation calls of three other species living in smaller colonies contained an estimate of 2 bits or less [12].

Isolation calls are a type of contact call. Hence, adult contact call variation should also correlate with social complexity. In some bat species, adults produce contact calls when held in isolation, searching for roosts, or alone in roosts with conspecifics calling outside [9], [13], [16], [17]. Adult bats can also eavesdrop on conspecific echolocation calls to find foraging or roosting locations, but the acoustic structure and directionality of biosonar pulses makes echolocation less ideal for intentional signaling [24] (but see [10], [25]). Social calls, by contrast, are typically longer in duration and lower in frequency, and hence travel farther with higher fidelity. Contact calls are likely to convey reliable information, such as individual identity or colony membership because they are often used to mediate cooperative interactions [9], [12]–[13], [18], [26]–[27].

There is evidence for heritable individual variation in bat social calls [28]–[29], but vocal learning can add another layer of complexity since contact calls might converge in structure between affiliated individuals [12], [30]–[35]. In the greater-spear nosed bat *Phyllostomus hastatus*, for example, newborn pups produce isolation calls with individual signatures [36], but female adults later join stable colonies and learn “screech calls”, which convey group, but not individual, identity [26]. These differences illustrate a match between structure and function, because individually distinct isolation calls allow recognition of each pup from 20 or so others, whereas the group-specific screech calls appear to coordinate group foraging [37].

In contrast to female greater spear-nosed bats, which live in stable social groups, other species demonstrate fission-fusion dynamics where individuals frequently switch roosts in such a way that groups split apart and recombine. Pallid bats (*Antrozous pallidus*), disc-winged bats (*Thyroptera tricolor*), noctules (*Nyctalus noctula*), Bechstein's bats (*Myotis bechsteinii*), and Natterer's bats (*Myotis nattereri*) are all species that switch roosts frequently and use contact calls to coordinate reunions at new roosting locations [16]–[19]. Here, contact calls with individual signatures allow particular individuals to roost together, even after foraging individually [18], [27].

**Figure 1:**
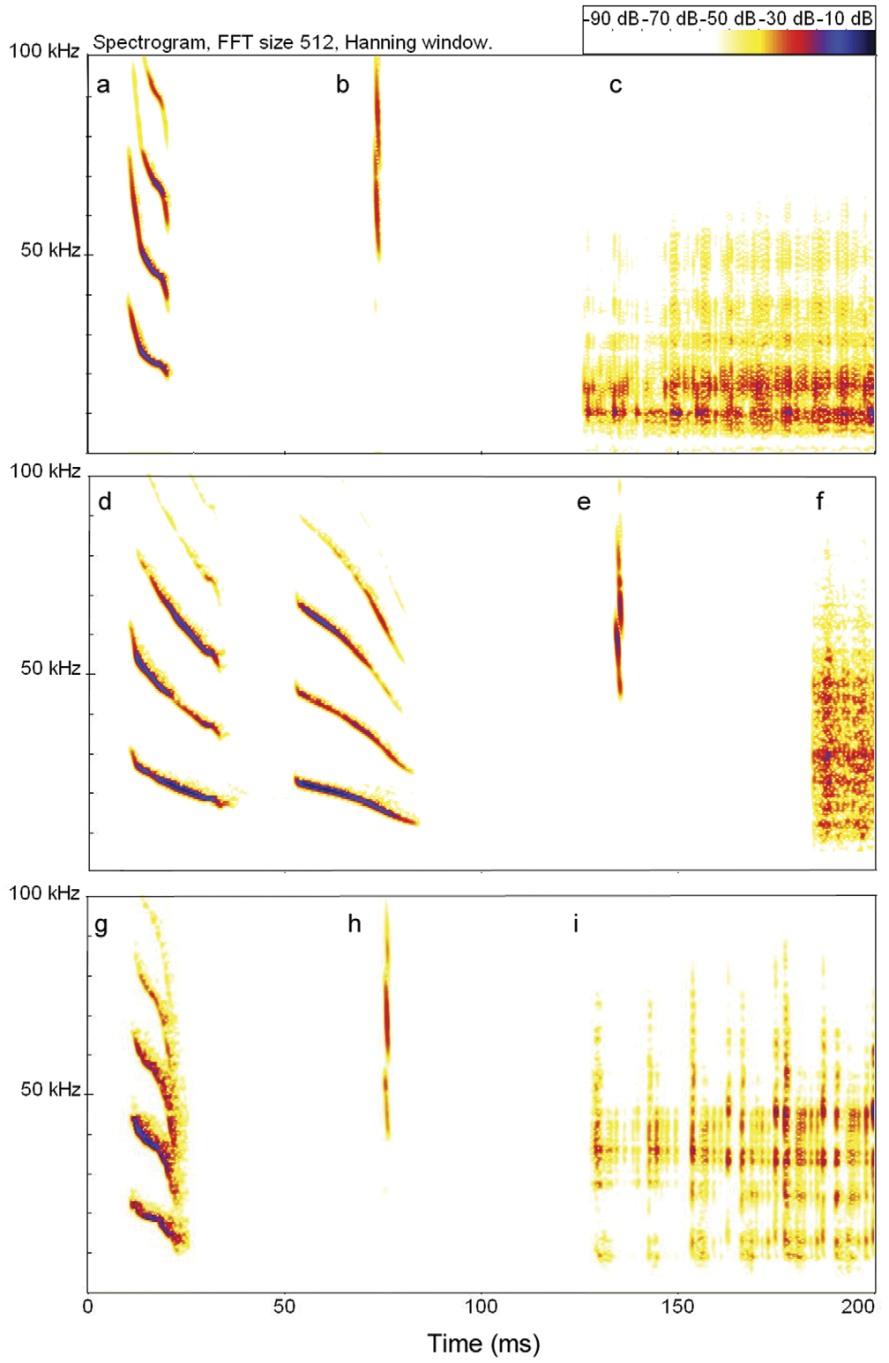
Spectrograms of calls from a common, white-winged, and hairy-legged vampire bat. Shown are a contact call (a), echolocation pulse (b), and portion of distress call (c) from a common vampire bat (*Desmodus rotundus*); double-note contact call (d), echolocation pulse (e), and portion of distress call (f) from a white-winged vampire bat (*Diaemus youngi*); contact call (g), echolocation pulse (h) and portion of a distress call (i) from a hairy-legged vampire bat (*Diphylla ecaudata*). Distress calls are often produced by captured bats.

The observation that social calls can mediate parental care [12], colonial roosting [16]–[18], group foraging [37], and collision avoidance [38] suggests that complex communication might mediate other cooperative social behaviors. Common vampire bats *Desmodus rotundus* show fission-fusion dynamics [12], [39], and possess the most cooperative social lives known among bats [12], [40]–[42]. *Desmodus* in Costa Rica roost in groups of 8–20 adults, which split apart and recombine, due to roost switching [39]. Females maintain long-term roosting affiliations that are largely independent of relatedness and last up to 12 years [12]. This species has been observed to survive 15+ years in the wild [43], with records twice as long in captivity [12], [44]. Such long-term social bonds involve cooperative behaviors such as allogrooming and regurgitated food sharing [39]–[41]. Unlike *Desmodus*, the social structures of the other two vampire species, the white-winged vampire bat (*Diaemus youngi*) and hairy-legged vampire bat (*Diphylla ecaudata*), have not been well studied in a natural context. However, all three species (Figure 1) are known to participate in social grooming and cooperative food sharing through regurgitation [13], [40], [45].

Adult *Diaemus* exchange individually distinct contact calls when isolated, and can use contact calls to vocally discriminate individuals [9], [13] and track their spatial locations [9]. *Desmodus* produce calls during both agonistic and affiliative interactions, and pups produce isolation calls that show variation between individuals [46]–[50]. However, no study has yet demonstrated individual signatures in adult *Desmodus* calls. To our knowledge, there are also no previous reports on social call variation in *Diphylla*.

Here we show that adults of all three species of vampire bat produce contact calls when physically, but not acoustically, isolated from conspecifics. We inspected contact call variation within and between the three vampire bat species using adult individuals recorded under the same conditions of social isolation. Our goals were to (1) describe contact call structure for each species, (2) test if contact calls contain information about individual identity, sex, colony, or population membership, and (3) determine if the vocal individuality of contact calls are encoded by similar or different call features.

## Methods

### Ethics Statement

We carried out our study in strict accordance with the recommendations in the Guide for the Care and Use of Laboratory Animals of the National Institutes of Health and National Research Council [51]. Captive bats were cared for by Organization for Bat Conservation (Cranbrook Institute of Science, Bloomfield Hills, Michigan), Rosamund Gifford Zoo (Syracuse, New York), or Talking Talons (Exhibitor's Permit no. 85-C-0021 issued by USDA Animal and Plant Health Inspection Service, Animal Care Division). Capture and temporary captivity of wild-caught vampire bats in Trinidad was approved by the University of Maryland Institutional Animal Care and Use Committee (Protocol R-10-63) and The Wildlife Section, Forestry Division, Republic of Trinidad and Tobago (Special Game License Permit). All fieldwork in Mexico was conducted under SEMARNAT DGVS permit # FAUT-0001.

### Recording procedure

All bats were caught in hand nets and placed individually in a small mesh cage for 1–24 hours within 10–30 cm of a CM 16 ultrasound condenser microphone (frequency range 10–200 kHz, Avisoft Bioacoustics, Berlin, Germany) and within hearing range of conspecifics. We digitized sounds with 16-bit resolution at a sampling rate of 250 kHz through an Avisoft Ultrasoundgate 116 or 416 on to a PC using Avisoft Recorder USG software.

### Acoustic analysis

We use the term “note” to describe a continuous element on a spectrogram (equivalent to “syllable” elsewhere [14], [18]). We restricted our analysis to the most common note type: a simple downward frequency-modulated sweep (e.g. Figure 1a). We analysed 2881 initial notes from five *Desmodus* from a captive colony at the Organization for Bat Conservation, 12 wild-caught *Desmodus* (caught in Trinidad, West Indies), 17 *Diaemus youngi* from a captive colony (New Mexico), one lone captive *Diaemus* (Syracuse), and three wild-caught *Diphylla ecaudata* (near Puebla, Mexico). We used these notes to obtain descriptive acoustic characteristics for each species and to calculate information capacity. For discriminant function analyses, we eliminated *Desmodus* and *Diaemus* individuals from which we did not record at least 45 notes, resulting in 2797 notes from four captive female *Desmodus*, four wild-caught female *Desmodus*, 18 captive *Diaemus*, and three wild-caught *Diphylla* (Table 1). We only analyzed notes that could be unambiguously assigned to individuals, except for notes from the three *Diphylla*, which we could not unambiguously assign to individual, and therefore pooled (Table 1).

**Table 1:** Vampire bats used for species-typical descriptions and information estimates.

Species	Population	Colony	Individual (age^a^)	Sex	Notes^b^	Tests^c^
*Desmodus rotundus*	MI, USA (captive)	A	Veronica (6)	F	249	1,2,4,5,7
		A	Bella (8)	F	217	1,2,4,5,7
		A	Vampirella (4)	F	201	1,2,4,5,7
		A	Lucy (6)	F	89	1,2,4,5
		A	Mya (16)	F	5	
	Trinidad (wild)	B	Dina	F	65	1,2,4,6
		C	Alice	F	60	1,2,4,6,7
		D	Wilkinsonia	F	51	1,2,4,6
		E	Cindy	F	45	1,2,4,6,7
		F	Angelica	F	37	
		n/a	Bianca	F	4	
		n/a	Cara	F	9	
		n/a	Ella	F	12	
		n/a	Fentonia	F	7	
		n/a	Bea	F	5	
		D	Dawkinsonia	F	1	
		D	MBF	F	4	
*Diaemus youngi*	NM, USA (captive)	G	Amber	F	70	1,2,3,8,9
		G	BeMary	F	90	1,2,3,8,9
		G	Cici	F	62	1,2,3,8,9
		G	Daniela	F	73	1,2,3,8,9
		G	Emily	F	62	1,2,3,8,9
		G	Farouk	M	68	1,2,3,8,9
		G	GaryMcCracken	M	72	1,2,3,8,9
		G	Hermanson	M	91	1,2,3,8,9
		G	Isaac	M	52	1,2,3,8,9
		G	JerryWilkinson	M	89	1,2,3,8,9
		G	Kristin	F	67	1,2,3,8,9
		G	Laurie	F	85	1,2,3,8,9
		G	MelvilleMerlin	M	51	1,2,3,8,9
		G	Nutella	F	69	1,2,3,8,9
		G	Oatmeal	M	55	1,2,3,8,9
		G	Punk	M	71	1,2,3,8,9
		G	RatcliffeRiskin	M	101	1,2,3,8,9
	NY, USA (captive)	H	Syracuse	M	65	1,2,3,8,9
*Diphylla ecaudata*	Mexico (wild)	I	3 bats pooled	M/F	527	1,2

a age in years at time of recording when known.

b number of calls analyzed (one note per call except Test 9);

c Indicates the permuted or conventional discriminant function analyses in which the individual bat was included. Test 1 is species assignment controlling individual (p = 0.001), and Test 2 is species assignment controlling colony (p = 0.001). Test 3 is sex assignment controlling individual (p = 0.7). Test 4 is population assignment controlling individual (p = 0.024). Test 5 is captive *Desmodus* individual assignment (p<0.001). Test 6 is wild *Desmodus* individual assignment (p<0.001), and Test 7 is individual assignment in *Desmodus* controlling recording session (p = 0.002). Test 8 is individual assignment of captive *Diaemus* using single notes (p<0.001), and Test 9 is individual assignment of captive *Diaemus* using double-notes (p<0.001). See results.

In our initial analyses, we included only the first note in a call, and excluded notes that occurred within 30 ms of a previous note. However, to analyze the effect of the double-note call structure commonly produced by *Diaemus,* we took measurements from both notes in a subsequent analysis. Only unclipped notes of adequate signal to noise ratio (10–99% amplitude) were included. We inspected spectrograms and oscillograms for each selected note in the program Batsound Pro (Pettersson Elektronik, Uppsala, Sweden). We hand-labeled the start and end of each note and used a custom-designed Matlab program [13] to automate 36 measurements between the start and end marks (Table 2), using 0.5 ms Blackman windows and 512-point FFTs (488 Hz resolution) with 50% overlap.

**Table 2:** Acoustic variables used for discriminant function analyses.

Variables	Explanation
Duration (ms)	Distance from start to end of note which we labeled by hand.
10 frequencies (Hz) along fundamental	Frequency measurements taken at the start, 10, 20, 30, 40, 50, 60, 70, 80, and 90 percent into the note. The end frequencies were removed because they often contained measurement errors, or were otherwise highly correlated with the frequency at the 90% mark.
4 frequencies (Hz) of most energy (FME)	Measured from fundamental and first 3 harmonics. Maximum values were calculated for the entire note.
4 times (ms) of FME	Measured from fundamental and first 3 harmonics. Time was measured relative to the start of the note.
7 slopes (kHz/ms) along fundamental	Frequency over time measurements taken at 20, 30, 40, 50, 60, 70, and 80 percent into the note.
7 concavities (kHz/ms/ms) along fundamental	Change in frequency over time measurements taken 20, 30, 40, 50, 60, 70, and 80 percent into the note.
3 intensities (dB) of a harmonic relative to fundamental	Intensity of 1^st^, 2^nd^, and 3^rd^ harmonic relative to the fundamental.

### General Statistical analysis

To assess the signature information capacity within contact calls, we estimated information in bits per signal, H_s_, obtained from an analyses of variance model using principal components extracted from call data [5]. Additionally, we calculated the probability of correct classification using conventional and permuted discriminate function analysis (DFA and pDFA) to train and test models. We favored this approach because it provides an empirical result (the rate at which new notes can be correctly classified) that is independent of model assumptions.

### Information calculation

Following Beecher [5] and Arnold & Wilkinson [18], we extracted principal components (PCs) with varimax rotation, then used restricted maximum likelihood to obtain the variance component estimate (VCE) of random factors (species, colony, individual) for each retained PC. We ran a parallel analysis [52] to determine how many PCs to extract from our data, and saved PC scores using the Bartlett method in IBM SPSS Statistics 19 (Chicago, IL, USA). To estimate the percentage of variance contributed by the random factors of species, colony, and individual, we weighted the VCE for each factor by the percentage variance explained by its corresponding PC.

We calculated the total signature information capacity of calls from the captive *Desmodus*, wild *Desmodus*, and captive *Diaemus* separately. We used the VCEs for colony and individual differences (S_B_^2^) and within-individual differences (S_T_^2^) to calculate the total variance (S_T_^2^), then summed the information in each PC, H_i_ = log_2_(S_T_/S_w_), to calculate total signature information in the call, H_s_, and the repeatability of each PC, S_B_^2^/(S_B_^2^+S_w_^2^) [5], [18].

### Conventional and permuted discriminant function analysis

We tested our ability to assign notes to individuals, colony, sex, or species using permuted discriminant function analyses (pDFA), a randomization approach that calculates confidence intervals for the observed classification rates with nested, non-independent data [53]. The pDFA performs two randomizations. First, it randomly selects training notes from each subject (e.g. individual bat) to derive linear discriminant functions, choosing the number of training notes such that an equal sample of notes is taken from each subject. The remaining unselected notes are then used for testing the discriminant functions (i.e. cross-validation). This DFA procedure is then repeated 100 times with different random selections from each subject to calculate a mean correct classification rate for the dataset [52].

In the second randomization step, 1000 randomized datasets are created where notes from the same subject (the control factor) remain together while a higher-level group label (the test factor), such as species, population, colony, or sex, is randomly shuffled and assigned to subjects. A DFA is performed with each of the 1000 shuffled datasets creating a distribution of correct assignment rates expected from random chance. The proportion of randomized datasets with a correct classification rate at least as large as the original dataset is a one-tailed p-value [53]. In summary, this resampling procedure provides a p-value for determining the significance of the observed correct classification rate of notes to the test factor (e.g. colony), while controlling for a single nested factor (e.g. individual).

Individual bats used in our analyses are shown in Table 1. To assess species-level differences, we conducted a pDFA to test our assignment of notes to the correct species controlling for individual (Test 1) or colony (Test 2). We tested assignment of notes of *Diaemus* to correct sex while controlling for individual (Test 3), and tested assignment of notes of female *Desmodus* to the correct population (captive and wild) controlling for individual (Test 4). Using conventional DFAs, we also tested for assignment of notes to correct individual within the captive *Desmodus* (Test 5) and wild *Desmodus* (Test 6). To test whether individual variation was merely due to differences between recording sessions, we also conducted a pDFA using five individuals (three captive and two wild *Desmodus*) for which we had an adequate sample of notes recorded from two different sessions 4–11 days apart (Test 7). Finally, we compared individual variation in captive *Diaemus* using tests with both single notes (Test 8) and double-notes (Test 9). We did not inspect individual variation in *Diphylla*, because we could not unequivocally match calls to individuals.

Conventional DFAs for individual signatures were conducted in SPSS using the “leave-one-out” procedure for cross-validation; Wilks' lambda was used to test the null hypothesis that notes from different individuals have equal mean discriminant scores, and a x^2^ approximation was used to obtain a p-value. Permuted DFAs [53] were conducted using a script written by Roger Mundry and implemented in R (The R Foundation for Statistical Computing, Vienna, Austria). We calculated 9 p-values from our DFA and pDFA tests, so we controlled our experimentwise error rate using a sequential Bonferroni correction [54]. Unless otherwise noted, correct classification rates are given for “test” notes (i.e. cross-validation) rather than for “training” notes used to build the model.

## Results

### Comparison of contact calls by species (Tests 1–2)

When isolated within audible range of conspecifics, adult females in all three species of vampire bats produced tonal calls similar but not identical to the contact calls previously recorded in *Diaemus youngi* [9], [13]. Female *Desmodus rotundus* produced mostly single-note calls; only 19% of notes were produced within 30 ms of a previous note (n = 2255). *Diphylla ecaudata* were similar; our three individuals produced only 13% of notes within 30 ms of a previous note (n = 608). In contrast, *Diaemus youngi* produced mostly double-note calls [13], [47]–[48], with a mean interval of 21.5 ms between notes (95% confidence interval, 21.0–22.0, n = 867). Out of 1456 *Diaemus* calls, 9% were single notes, 75% were double notes, and 16% were three or more notes. We observed antiphonal exchanges between adult *Diaemus* but not between adults of the other two species.

Note structure varied by species (Figure 1, Table 3). Using only the first notes from calls, we could assign 94% of test notes to the correct species when controlling for individual (Test 1, pDFA, n = 2023, p = 0.001) or 93% of test notes when controlling for colony (Test 2, pDFA, n = 2689, p = 0.001). For training notes used to construct the discriminant functions, the corresponding correct classification rates were 96% and 97% respectively (n = 1073, 407). Overall, *Desmodus* produced the shortest and steepest calls, typically single notes that were the highest in frequency, with first and second harmonics carrying more energy than the fundamental. *Diaemus* produced the longest and least steep calls, typically double notes that were intermediate in frequency, with the fundamental carrying more energy than harmonics. *Diphylla* produced the lowest frequency calls of intermediate duration and slope, with the fundamental carrying less energy than the harmonics.

**Table 3:** Mean±standard error for 12 acoustic variables by species.

Acoustic Variable	*Desmodus*	*Diaemus*	*Diphylla*
Duration (ms)	9.1±0.1	18.6±0.2	11.5±0.1
Fundamental frequency (kHz) at start	36.2±0.2	25.7±0.1	20.8±0.2
Fundamental frequency (kHz) 50% into note	23.4±0.1	20.9±0.1	16.5±0.1
Fundamental frequency (kHz) 90% into note	19.3±0.1	17.3±0.1	12.6±0.1
Frequency of most energy (kHz)	25.4±0.1	22.1±0.1	16.4±0.2
Time of frequency of most energy (ms)	3.7±0.1	7.4±0.1	6.3±0.2
Slope of fundamental (kHz/ms) 10% into note	−6.2±0.3	−2.2±0.0	−3.6±0.1
Slope of fundamental (kHz/ms) 50% into note	−2.1±0.0	−0.6±0.0	−0.9±0.0
Slope of fundamental (kHz/ms) 90% into note	1.1±0.1	1.2±0.1	1.9±0.1
Amplitude of first harmonic relative to fundamental (dB)	6.6±0.2	−2.8±0.3	9.9±0.3
Amplitude of second harmonic relative to fundamental (dB)	6.6±0.3	−4.3±0.5	9.7±0.6
Amplitude of third harmonic relative to fundamental (dB)	−1.1±0.4	−18.8±0.4	1.0±0.6

### Sex and population-level variation in note structure (Tests 3–4)

While controlling for individual variation, we found no significant effect of sex on the note structure of the captive *Diaemus* (Test 3, n = 375, p = 0.7). We demonstrated population-level variation in *Desmodus* notes by correctly classifying 87% of training notes (n = 333) and 83% of test notes to the correct wild or captive population while controlling for individual variation (Test 4, n = 675, p = 0.024).

### Individual signatures (Tests 5–9)

**Figure 2:**
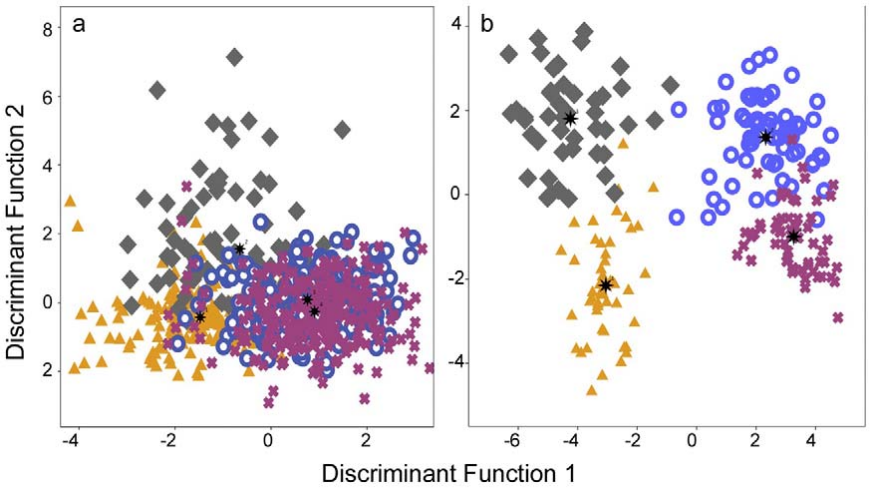
Vocal individuality shown by the overlap in discriminate scores of calls from four common vampire bats from either a single captive colony (a) or four different wild colonies (b). Plot shows the discriminant scores for the first two canonical discriminant functions constructed separately for each population. The four bats in each plot are denoted by different symbols.

*Desmodus* contact calls could be assigned to individuals with greater than chance accuracy (Table 4). Furthermore, wild individuals from different colonies produced calls that were more individually distinct than bats from the same captive colony; the mean classification rate was significantly higher in the wild-caught bats from different colonies than the bats from a single captive colony (t = 2.987, df = 6, p = 0.024, Table 4, Figure 2). The overall correct classification rate was 66% for four bats from a single captive colony (Test 5, Wilks' lambda = 0.27, x^2^ = 948.0, df = 126, N = 756, p<0.001, Table 4) and 91% for four wild-caught bats from different colonies (Test 6, Wilks' lambda = 0.01, x^2^ = 955.6, df = 153, N = 221, p<0.001, Table 4). The ability to assign notes to correct individuals remained after controlling for recording session (Test 7, pDFA, n = 682, p = 0.002).

**Table 4:** Percentage of correctly assigning notes to four individuals for captive and wild common vampires *Desmodus rotundus* and captive white-winged vampires *Diaemus youngi*.

Population	Colony	Bat	Correct classification rate (chance = 25%)	
			Training notes	Testing notes
captive *Desmodus*	A	Veronica	67%	63%
	A	Bella	87%	82%
	A	Vampirella	69%	65%
	A	Lucy	40%	37%
**wild ** ***Desmodus***	**B**	**Dina**	**97%**	**88%**
	**C**	**Alice**	**100%**	**98%**
	**D**	**Wilkinsonia**	**98%**	**94%**
	**E**	**Cindy**	**100%**	**84%**
captive *Diaemus*	G	Amber	93%	81%
	G	BeMary	97%	94%
	G	Cici	92%	84%
	G	Daniela	90%	88%

In *Diaemus* contact calls, the first notes were individually variable (Test 8, Wilks' lambda = 0.02, x^2^ = 4967.1, df = 612, N = 1293, p<0.001). For first notes, the overall correct classification rate was 57% (chance rate = 6%, range = 11–87%). However, when we included measurements from both notes, the overall correct classification rate increased to 72% (Test 9, range = 48–90%, Wilks' lambda = 0.001, x^2^ = 6034.3, df = 867, N = 866, p<0.001).

### Information analysis and variation within individuals

Contact calls were also highly variable within individuals. Six extracted principal components accounted for 74% of the total variance, and of this variation, 40.9% was accounted for by species (16.4%), colony (8.5%) and individual (16.0%) differences. The low stereotypy and high variability of notes within calls of individuals were reflected by relatively low estimates of signature information (*Diaemus* = 3.21, captive *Desmodus* = 3.26, wild *Desmodus* = 3.88 bits) and low estimates of repeatability across PCs (*Diaemus*: mean = 0.15, range = 0.004–0.444; captive *Desmodus* mean = 0.14, range = 0.014–0.26; wild *Desmodus*: mean = 0.34, range = 0.15–0.57). For the subfamily Desmodontinae as a whole, contact calls contained 4.4 bits with a mean repeatability of 0.31 (range = 0.09–0.70). Despite the within-individual variation, we were still able to assign notes to correct species, populations, and individuals with high accuracy.

### Acoustic parameters encoding individual variation

A summary of the canonical structure matrix for the DFA on individual variation in *Desmodus* notes shows that the acoustic parameters defining the first discriminant function differed between the captive and wild populations (Table 5). In notes from the population of wild individuals from different colonies, all of the five highest loading parameters were measures of frequency. By contrast, in notes from the captive colony, the highest loading parameter was the relative intensity of the third harmonic.

**Table 5:** Highest variable loadings for discriminant functions assigning notes to individual common vampire bats.

Captive bats, same colony		Wild bats, different colonies		
**Discriminant Function 1**				
Relative intensity 3rd harmonic	−0.334		Frequency 90% into note	0.631
Frequency 90% into note	0.319		Frequency 80% into note	0.579
Time of FME^a^ in 2nd harmonic	0.302		Frequency 70% into note	0.481
Frequency 80% into note	0.300		FME of 3rd harmonic	0.431
Relative intensity 1st harmonic	0.295		FME of 2nd harmonic	0.341
**Discriminant Function 2**				
Slope 20% into note	−0.450		Slope 20% into note	0.627
Slope 30% into note	−0.432		Slope 30% into note	0.559
Concavity 50% into note	0.413		Slope 40% into note	0.525
Slope 40% into note	−0.405		Slope 50% into note	0.470
Slope 50% into note	−0.362		Duration	0.441
**Discriminant Function 3**				
FME of 2nd harmonic	0.430		Frequency 10% into note	0.490
Time of FME in 2nd harmonic	−0.428		Frequency 20% into note	0.444
Duration	−0.361		Frequency 30% into note	0.379
Frequency 70% into fundamental	0.352		Concavity 70% into note	−0.374
Time of FME in 1st harmonic	−0.344		Slope 80% into note	−0.345

a frequency of most energy

For each discriminant function, the 5 variables with the highest pooled within-colony correlations with the standardized discriminant functions are shown.

A summary of the canonical structure matrix for the DFA on individual variation in *Diaemus* notes (Table 6) show that discriminate function 1 was largely defined by the starting frequencies, discriminant function 2 was highly correlated with duration, discriminant function 3 was highly correlated with timing and frequency of the frequency of most energy in the fundamental, and discriminant function 4 was largely defined by the ending frequencies. These four discriminant functions explained 72.7% of the total variance.

**Table 6:** Highest variable loadings for discriminant functions assigning notes to individual white-winged vampire bats.

First note only		Both notes in double-note calls^a^		
**Discriminant function 1**				
Frequency at 30% into note	0.733		Frequency at 10% into note 2	0.450
Frequency at 20% into note	0.732		Frequency at 10% into note 1	0.412
Frequency at 10% into note	0.666		Frequency at 50% into note 1	0.394
Frequency at 40% into note	0.650		Frequency at 90% into note 2	0.317
Frequency at 50% into note	0.530		Frequency 50% into note 2	0.298
**Discriminant function 2**				
Duration	0.795		Frequency of most energy of the fundamental of note 2	−0.457
Frequency at 80% into note	−0.596		Frequency at 10% into note 2	−0.434
Frequency at 70% into note	−0.567		Time of the FME of fundamental in note 1	0.393
Time of the FME of third harmonic	0.536		Duration of note 1	0.345
Frequency at 90% into note	−0.475		Time of the FME of fundamental in note 2	0.320
**Discriminant function 3**				
Time of the frequency of most energy of the fundamental	0.520		Frequency at 10% into note 1	−0.479
Frequency of most energy of the fundamental	−0.412		Duration of note 1	0.355
Frequency at 10% into note	0.377		Interval between notes (ms)	0.336
Time of the frequency of most energy of the third harmonic	0.314		Slope 50% into note 1	0.291
Frequency at start of the note	0.288		Slope 40% into note 1	0.285
**Discriminant function 4**				
Frequency at 70% into note	0.523		Slope 50% into note 2	0.497
Frequency at 60% into note	0.511		Slope 60% into note 2	0.462
Frequency at 80% into note	0.470		Slope 40% into note 2	0.371
Frequency at 50% into note	0.437		Slope 70% into note 2	0.367
Frequency at 90% into note	0.379		Duration of note 1	−0.355

a This dataset is missing values for frequencies at 20,30,40,70, and 80% into notes 1 and 2. Interval between notes (ms) is an additional variable.

For each discriminant function, the 5 variables with the highest pooled within-colony correlations with the standardized discriminant functions are shown.

## Discussion

### Variation in contact calls

Contact calls of vampire bats (Phyllostomidae: Desmodontinae) were highly variable between individual bats, even when considering only the first notes of downward sweeping tonal calls. Our acoustic analyses also show that the variation of contact calls produced by a single isolated bat was quite high with 59% of the variation explained by our extracted principal components occurring within individuals. Species and individual differences made roughly equivalent contributions to total variation in contact calls, while population and group factors contributed less variation. In summary, all three vampire bats produced contact calls that were not completely stereotyped within individuals, but demonstrated individual signatures when we tested for them using permuted and conventional discriminant function analyses.

The estimated signature information capacity of the adult vampire bat contact calls in our study was greater than the estimated information in the pup isolation calls of the following bat species (listed in increasing information content): *Nycticeinops schlieffenii* (non-colonial), *Cleotis percivali* (colonial), *Scotophilus borbonicus* (colonies of up to 100 bats), and *Rhinolophus simulator* (colonies of several hundred). Adult vampire bat contact calls contained less signature information than isolation calls of *Hipposideros caffer* (colonies of thousands), *Chaerephon pumila* (colonies of 20–500 bats), *Nycticeius humeralis* (colonies up to 1000 bats), and *Tadarida brasiliensis* (colonies over a million) [12], and also less than the adult contact calls of *Antrozous pallidus* (colony size at study site of up to 100 bats) [18]. *A. pallidus* produced contact calls with greater stereotypy within an individual across days [18], whereas *Desmodus* calls from a single bat in a single context were highly variable.

### Individual signatures in the common vampire bat *Desmodus rotundus*

Both the captive and wild *Desmodus* in our study conveyed individual identity in their contact calls, and several lines of evidence suggest that common vampires can vocally discriminate individuals. First, previous studies [9], [13] demonstrated that the closest extant taxa, *Diaemus youngi*, which possess similar individual signatures in their contact calls, can vocally discriminate individuals. This was revealed by habituation-discrimination playback experiments [13], and by the experimental result that shuffling the relative positions of caged bats to new locations leads to more calling and a higher response rate compared to shuffling bats back to the same locations [9]. Anecdotal observations also suggest that highly associated individual *Desmodus* are attracted to each other's contact calls. Captive individuals released back into cages were immediately attended to by roostmates known to have high levels of relatedness or past association (GGC, unpublished data). In another case, a captured wild bat was released and flew out of a building, but when a second bat caught at the same location began calling, the first bat re-entered the building and hung on the ceiling nearby producing social calls. Finally, *Desmodus* are able to recognize the breathing sounds of individual humans [55], which highlights their ability to use subtle and complex acoustic cues for vocal recognition.

### Double-note contact calls of the white-winged vampire bat *Diaemus youngi*

*Diaemus youngi* are the most vocal of the vampire bats. They produce more calls, their calls have more notes, and their notes are longer and likely more individually distinct than the other two bat species. To record contact calls from *Desmodus* and *Diphylla*, we left bats in physical isolation for up to 24 hours, but *Diaemus* often produced more than 50 calls in less than two hours. In many bats, including *Desmodus*, mothers and pups exchange social calls. However, we only observed adult antiphonal exchanges in *Diaemus*, where calls are exchanged with a latency of about 1/3^rd^ of a second [13].

Contrary to our expectations, the single *Diaemus* individual from a different population produced notes that were not more distinct. In fact, the correct classification rate for this individual (11%) was an outlier for poor accuracy of classification (n = 18, chi-square outlier test: X^2^ = 5.0364, p = 0.025) when considering only the first note of a call. However, when considering both notes of a call, this discrepancy disappeared, suggesting that the second note in this individual contained much of the signature information. This observation again highlights the fact that double-note call structures allow for substantial increases in potential information content.

The acoustic parameters defining vocal individuality differed between *Desmodus* (Table 5) and *Diaemus* (Table 6). Individuality in *Desmodus* notes was largely explained by minimum frequencies, slopes, and, in the captive bats, harmonic structure. Whereas in the captive *Diaemus* calls, discriminant function 1 was defined largely by the frequency values of the frequency of the first half of the note. Discriminant function 2 was most highly correlated with duration. Discriminant function 3 was largely correlated with the frequency of most energy and its timing. Discriminant function 4 was defined largely by the last half of the note.

### Population and colony variation in common vampire bats *Desmodus rotundus*

After controlling for individual as a factor, we could correctly assign notes to the wild-caught Trinidad population or the captive population with greater than chance accuracy. Interestingly, we could more easily assign notes to wild-caught bats from different colonies than to the bats from a single captive colony (Table 4, Figure 2). Calls from wild-caught vampire bats encoded more signature information (3.9 bits wild versus 3.3 bits captive). In other words, vampire bats in the captive colony produced calls with less vocal individuality compared to wild bats from different colonies. These acoustic differences might result from a number of possible factors, including long-term captivity, geographic variation, or vocal convergence in colonies [56]. However, the greater call similarity in the captive colony is not likely to be explained by genetic relatedness. We estimated the pairwise relatedness of individuals from both populations using 13 microsatellite loci [57]–[58], and found that the four individuals used in this study appear genetically unrelated to each other as are the four wild *Desmodus* caught at different sites.

Vocal learning in bats has been demonstrated when pup calls converge with mothers [30]–[32] or adult males [35], and when adult *Phyllostomus hastatus* learn group-specific calls [33]. If the captive *Desmodus* vocally converged in call structure, we might expect differences in the acoustic parameters encoding individuality. We predicted that vocal learning would be more likely to change sound production at the larynx (which primarily determines duration, frequency, and frequency modulation of the fundamental) than at the vocal tract (which determines the relative intensity of harmonics), because the vocal folds in the larynx are relatively plastic while the vocal tract above the vocal cords is stable by comparison [59]. Put differently, we predicted that vocal learning in the captive population would have led to converge among the laryngeal-based parameters such as frequency rather than harmonic structure, which is shaped by vocal tract filtering. Consistent with this expectation, the vocal individuality of the wild bats from different groups was based relatively more on differences in the frequencies and modulation of the fundamental, whereas vocal individuality in the captive colony relied relatively more on the distribution of energy across harmonics (Table 5). Further study is needed to determine if vampire bats are capable of vocal learning.

### Function of contact calls in adult vampire bats

Pup isolation calls have a clear function, but there is much we do not understand about the function of adult contact calls in vampires or other bat species. It remains unclear why adult vampire bats produce contact calls when isolated. Future work should address whether highly affiliated individuals are more likely to respond to contact calls than other conspecifics and if calls produced in other social contexts (e.g. allogrooming, food sharing) have the same or different structure. Finally, we do not yet know if the large variation produced by a single isolated individual bat corresponds to random variation or unidentified social factors such as the caller's motivational state, perceived proximity to conspecifics [60], or stress [61]–[64].
